# Supplementary material for: PIFiA: self-supervised approach for protein functional annotation from single-cell imaging data
Source: Mol Syst Biol. 2024 Mar 12;20(5):521–48. doi: 10.1038/s44320-024-00029-6 (PMC11066028; doi:10.1038/s44320-024-00029-6)
Supplement: Supplementary file 1 — Appendix [file 44320_2024_29_MOESM1_ESM.pdf]

Appendix Materials

Manuscript Title

PIFiA: Self-supervised Approach for Protein Functional Annotation from Single-Cell Imaging Data

Authors

Anastasia Razdaibiedina[1,2,4], Alexander Brechalov[1,2], Helena Friesen[2], Mojca Mattiazzi Usaj[2*], Myra Paz David Masinas[2], Harsha Garadi Suresh[2], Kyle Wang[1,2], Charles Boone[1,2,5†], Jimmy Ba[3,4,†], Brenda Andrews[1,2,†]

[1] Department of Molecular Genetics, University of Toronto, Toronto ON, Canada
[2] The Donnelly Centre, University of Toronto, Toronto ON, Canada
[3] Department of Computer Science, University of Toronto, Toronto ON, Canada
[4] Vector Institute for Artificial Intelligence, Toronto ON, Canada
[5] RIKEN Center for Sustainable Resource Science, 2-1 Hirosawa, Wako, Saitama, Japan.
*Current address: Department of Chemistry and Biology, Toronto Metropolitan University, Toronto, ON, Canada

Corresponding authors

Brenda Andrew
Email: brenda.andrews@utoronto.ca

Jimmy Ba
Email: jba@cs.toronto.edu

Charles Boone
Email: charlie.boone@utoronto.ca

Table of Contents

Appendix Contents **Page Number**

Appendix Figure S1……………………………………………………………………………………………………3

Appendix Figure S2……………………………………………………………………………………………………5

Appendix Figure S3……………………………………………………………………………………………………6

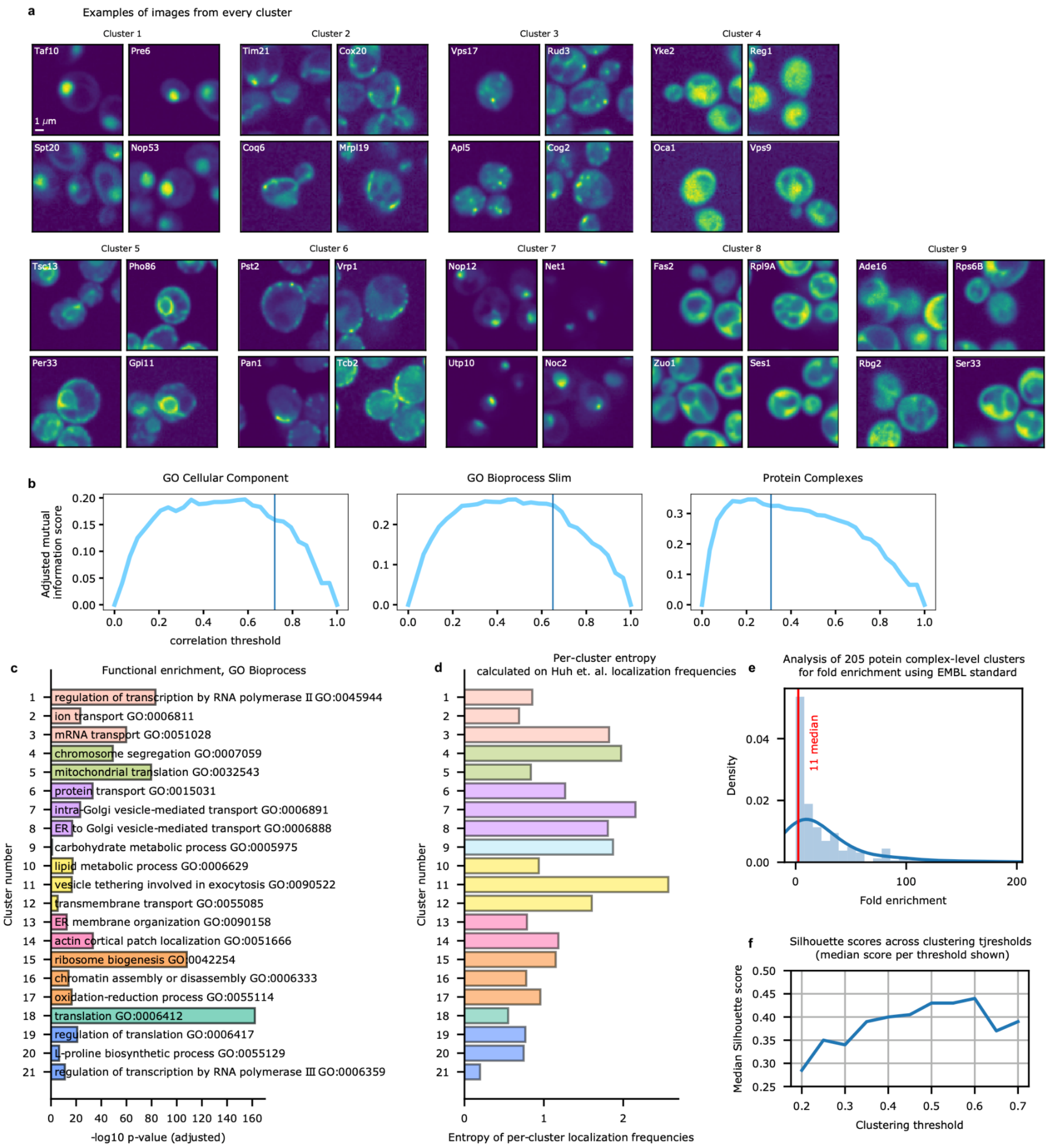

Appendix Figure S1. Examples of AMI cutoffs and cell images from nine clusters. Related to Fig 3.

- **A.** Examples of GFP-tagged proteins from nine clusters corresponding to major cellular components.
- **B.** Plots showing adjusted mutual information across different correlation thresholds for cellular component, bioprocess and protein complex standards. Vertical line indicates a point of a dendrogram cut determined by saturation of a score.
- **C.** Bar graphs showing the top Gene Ontology Biological Process terms and corresponding functional enrichments for 21 clusters obtained from clustering by GO Biological Process Slim cutoff.

D. Bar graph showing entropy across 21 bioprocess clusters calculated from localization category frequencies (from Huh et al. (2003) standard).

E. Distribution of fold enrichments on EMBL protein complex standard for 205 clusters derived from Protein Complex cutoff.

F. Silhouette scores for different thresholds across single-localizing aFPs from the same localization category.

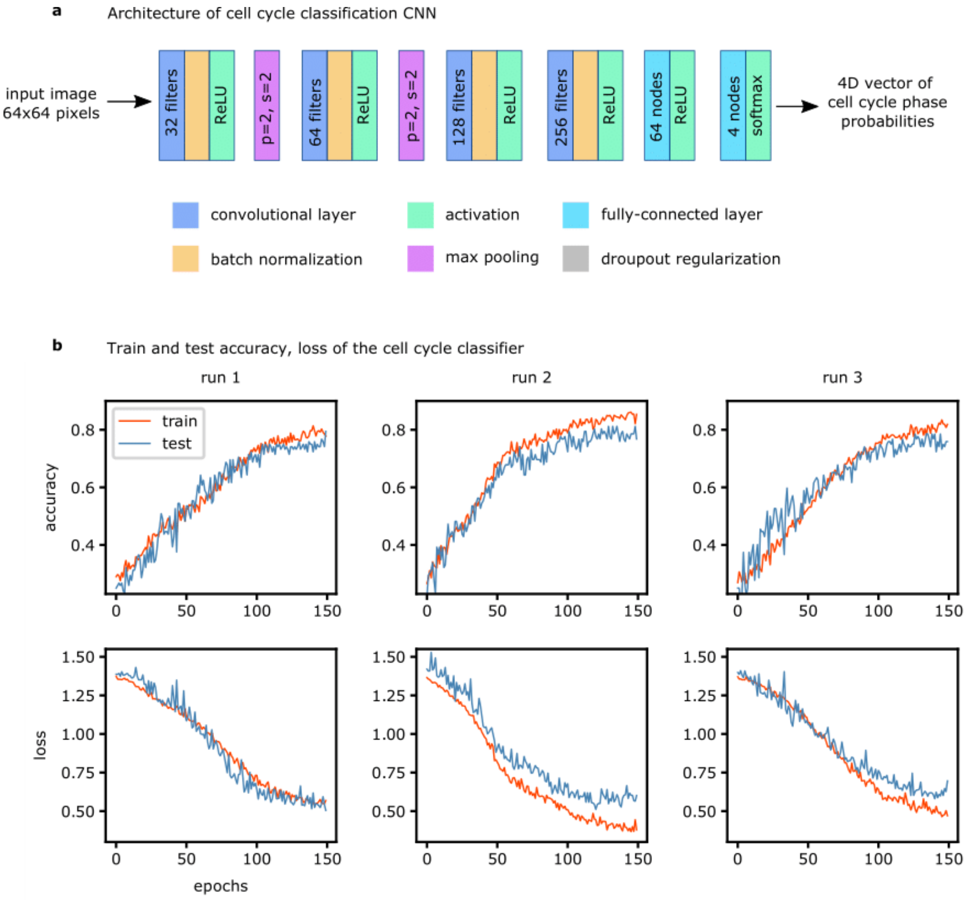

Appendix Figure S2. Cell cycle classifier training settings. Related to Fig 4.

A. Architecture of the convolutional neural network used for cell cycle classification.
B. Train and test set performance (accuracy and loss) of the cell cycle classifier across three independent network runs.

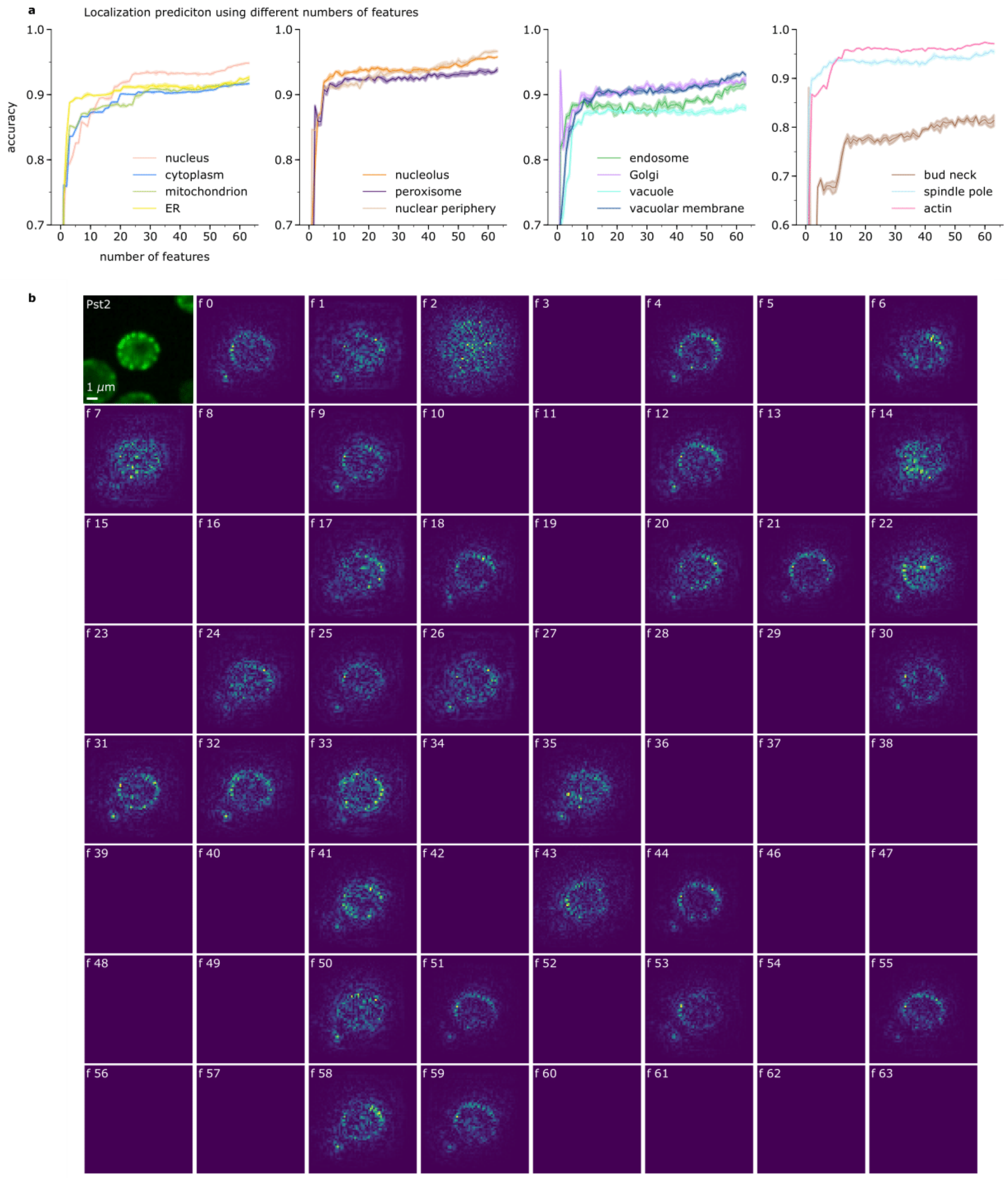

Appendix Figure S3. Extended interpretability studies. Related to Fig 6.

A. Plot of accuracy of subcellular localization prediction as a function of dimensionality of feature profiles.
B. Examples of feature-wise gradient maps obtained with SmoothGrad for all features of Pst2-GFP protein crop.
