# Supplementary material for: PIFiA: self-supervised approach for protein functional annotation from single-cell imaging data
Source: Mol Syst Biol. 2024 Mar 12;20(5):521–48. doi: 10.1038/s44320-024-00029-6 (PMC11066028; doi:10.1038/s44320-024-00029-6)
Supplement: Supplementary file 7 — Expanded View Figures [file 44320_2024_29_MOESM7_ESM.pdf]

Expanded View Figures

Figure EV1. PIFiA network architecture and training settings.

Related to Fig. 1. (**A**) Overview of the architecture of PIFiA convolutional network. (**B**) Left plot: test accuracies of three different runs over the course of training (X axis: epochs, Y axis: test accuracy). Smaller plots: average precision, F-score and precision on protein complexes and pathways standards (X axis: epochs, Y axis: corresponding score on test set). The purple line indicates point of early stopping, when accuracy starts to saturate (derivative of the test accuracy smaller than a threshold of 0.5%). (**C**) Bar graphs comparing the current PIFiA architecture with a common baseline, DenseNet-121, across four different standards (Gene Ontology Cellular Component, Gene Ontology Bioprocess Slim, KEGG Pathways, EBI Protein complexes) in terms of average precision, F-score and adjusted mutual information (assessed on aFPs of 4049 proteins). Error bars represent standard deviation from the mean across three network runs. (**D**) Bar graphs comparing PIFiA performance across different dimensions of the feature profiles (32, 52, 64, 80, 128).

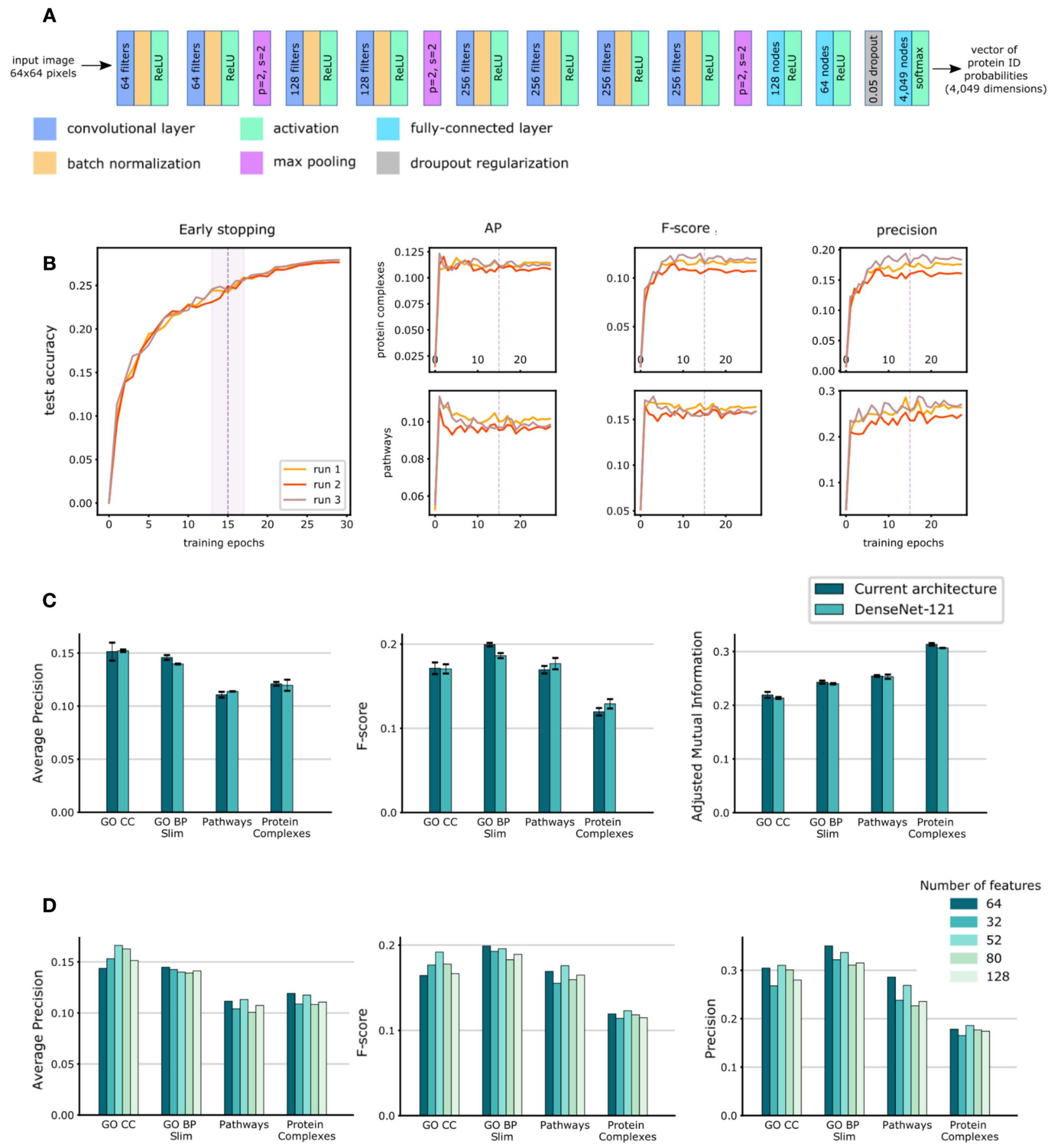
A
input image 64x64 pixels
64 filters
ReLU
64 filters
ReLU
p=2, s=2
128 filters
ReLU
128 filters
ReLU
p=2, s=2
256 filters
ReLU
256 filters
ReLU
256 filters
ReLU
256 filters
ReLU
p=2, s=2
128 nodes
ReLU
64 nodes
ReLU
0.05 dropout
4,049 nodes
softmax
vector of protein ID probabilities (4,049 dimensions)
convolutional layer
activation
fully-connected layer
batch normalization
max pooling
droupout regularization
B
Early stopping
test accuracy
training epochs
run 1
run 2
run 3
AP
F-score
precision
protein complexes
pathways
training epochs
C
Current architecture
DenseNet-121
Average Precision
F-score
Adjusted Mutual Information
GO CC
GO BP Slim
Pathways
Protein Complexes
D
Number of features
64
32
52
80
128
Average Precision
F-score
Precision
GO CC
GO BP Slim
Pathways
Protein Complexes

 © The Author(s)

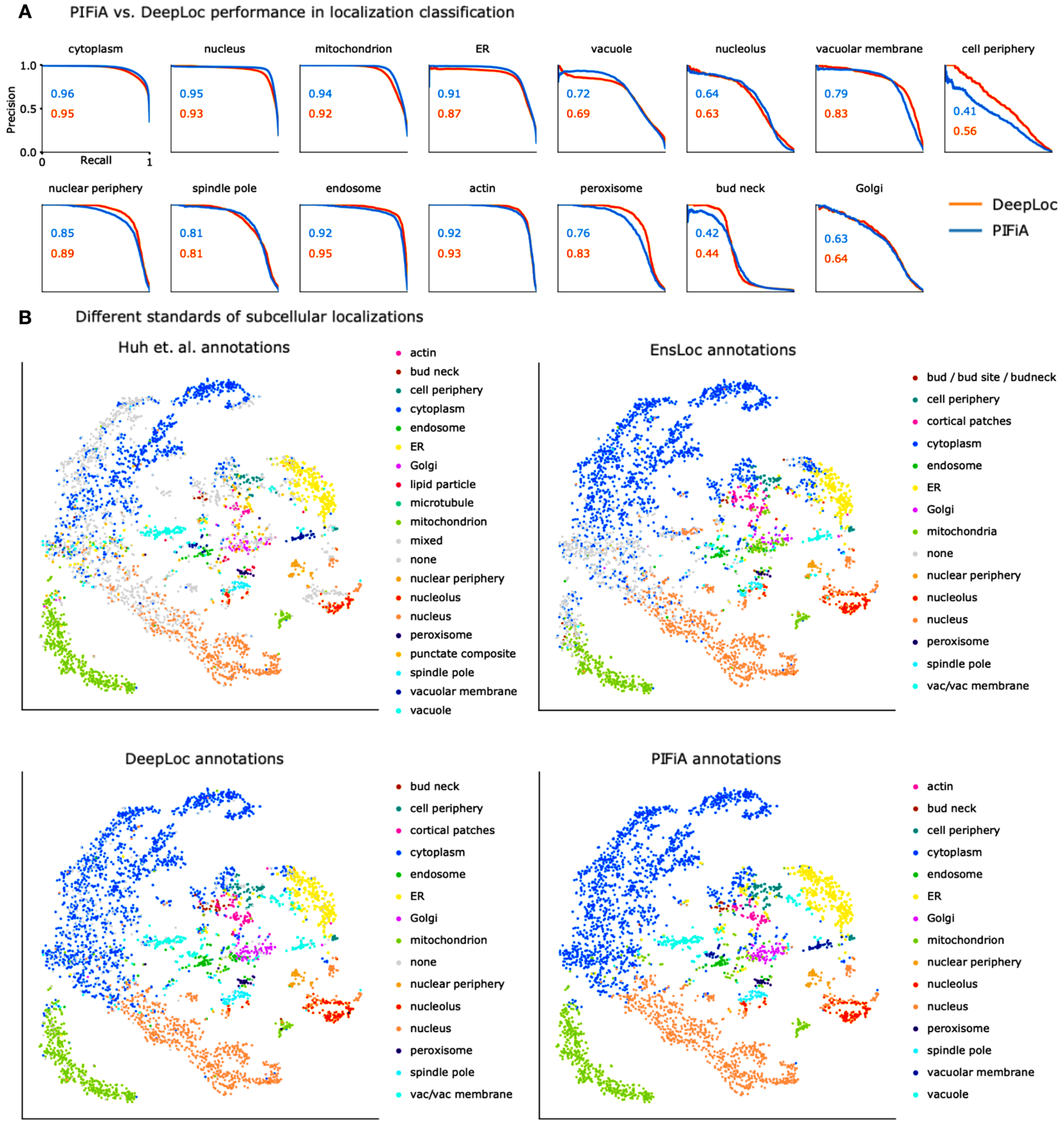

Figure EV2. Comparison of PIFiA annotations and existing localization standards.

Related to Fig. 3. (**A**) Comparison of localization classification performance of DeepLoc versus PIFiA feature profiles coupled with a logistic regression. Precision-recall plots are shown for 15 subcellular localizations. (**B**) Whole-proteome aFPs tSNE colored by different annotations of subcellular localization: manual annotations from Huh et al, (2003) (Huh et al, 2003), and computationally-derived annotations from EnsLoc, DeepLoc and PIFiA.

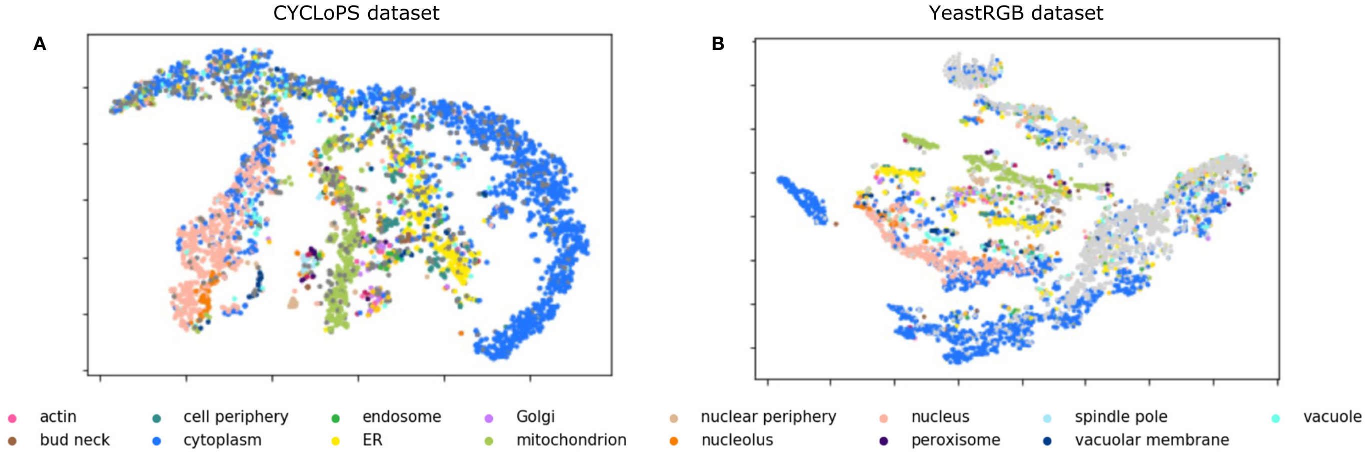

Figure EV3. Generalization of PIFiA network to two unseen datasets.

Related to Fig. 3. (**A**) Localization-colored tSNE on aFPs obtained from the CYCLoPS dataset. (**B**) Localization-colored tSNE on aFPs obtained from the YeastRGB dataset.

 © The Author(s)

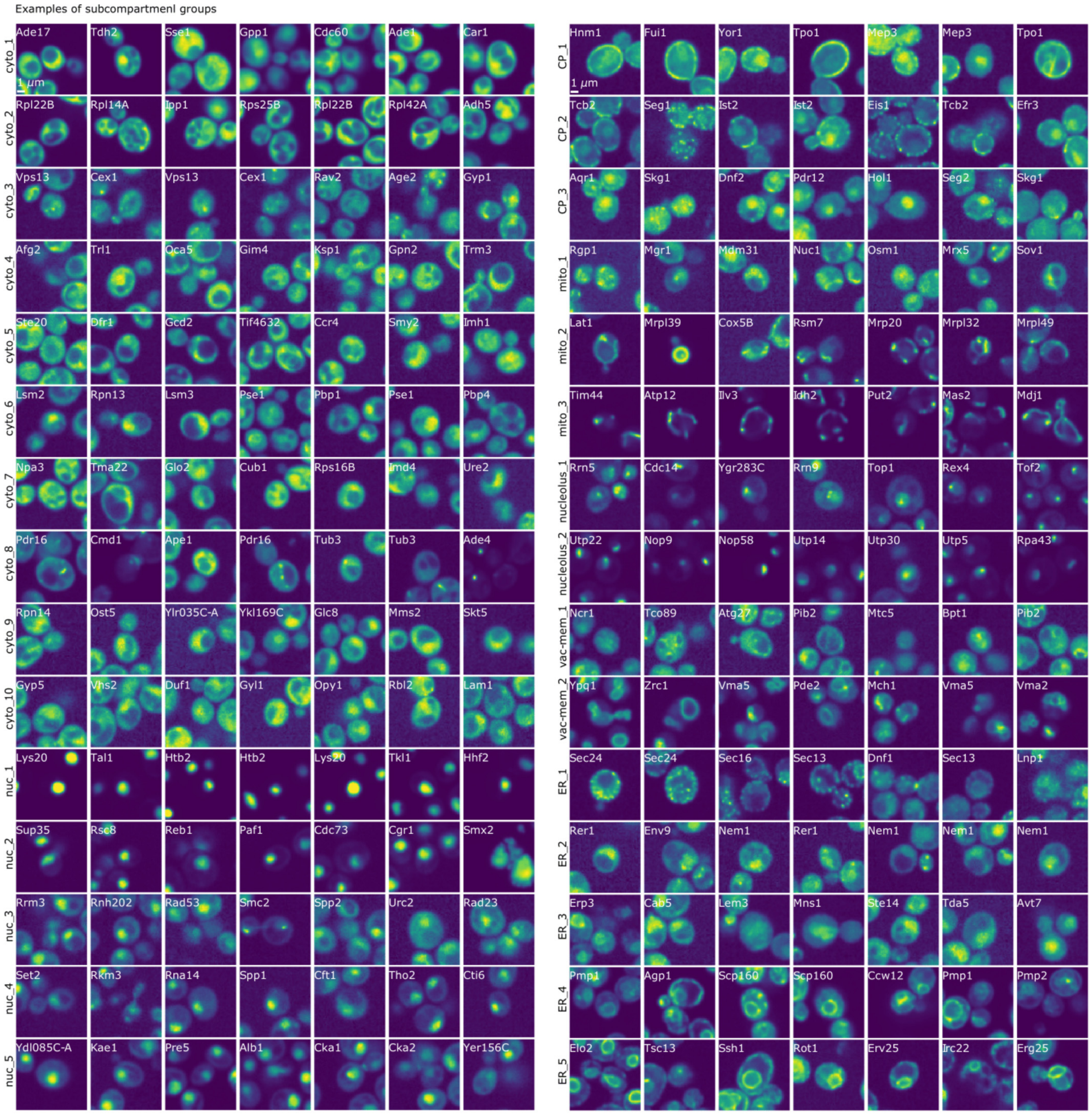

Figure EV4. Examples of proteins from 30 different sub-compartmental groups.

Related to Fig. 3. Each row corresponds to a sub-compartmental cluster (e.g. nuc-1, nuc-2). The relevant GFP-tagged protein is identified on each micrograph.

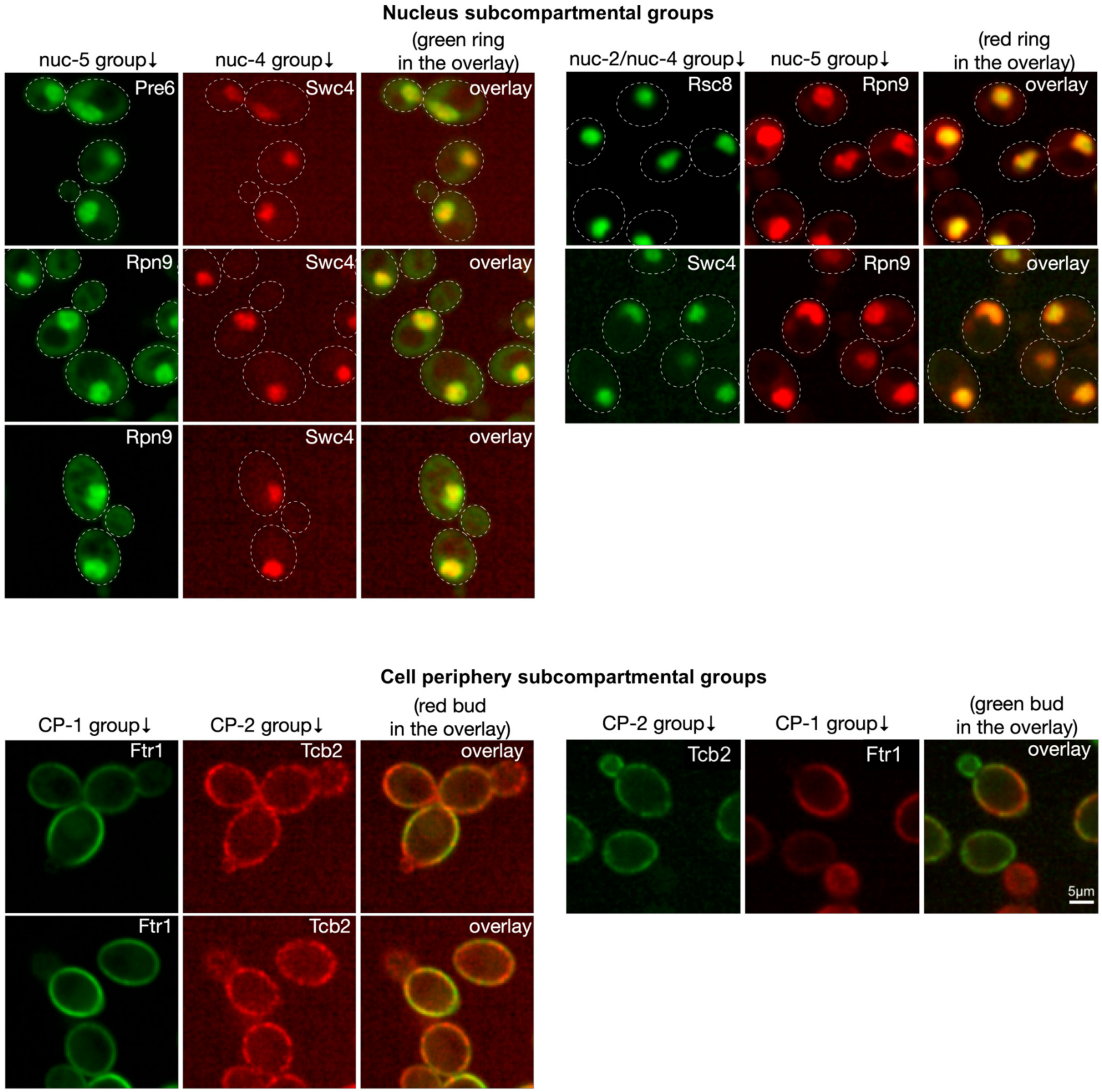

Figure EV5. Colocalization assay for proteins from different sub-compartmental.

Related to Fig. 3. Colocalization experiment results: representative micrographs of cells expressing mNeonGreen- (green images) or mScarlet- (red images) tagged proteins annotated to nucleus (top panel) or cell periphery (bottom panel) groups. Overlays of the mNeonGreen and mScarlet images are shown on the right of each triplet of images. The tagged proteins are indicated on the micrographs (scale bar shown bottom right).

 © The Author(s)
